# Antioxidant Activity Mediates Pirfenidone Antifibrotic Effects in Human Pulmonary Vascular Smooth Muscle Cells Exposed to Sera of Idiopathic Pulmonary Fibrosis Patients

**DOI:** 10.1155/2018/2639081

**Published:** 2018-10-21

**Authors:** Alessandro Giuseppe Fois, Anna Maria Posadino, Roberta Giordo, Annalisa Cossu, Abdelali Agouni, Nasser Moustafa Rizk, Pietro Pirina, Ciriaco Carru, Angelo Zinellu, Gianfranco Pintus

**Affiliations:** ^1^Department of Clinical and Experimental Medicine, University of Sassari, Viale San Pietro 43, 07100 Sassari, Italy; ^2^Department of Biomedical Sciences, University of Sassari, Viale San Pietro 43, 07100 Sassari, Italy; ^3^Biomedical Research Center, Qatar University, 2713 Doha, Qatar; ^4^Pharmaceutical Science Section, College of Pharmacy, Qatar University, 2713 Doha, Qatar; ^5^Department of Biomedical Sciences, College of Health Sciences, Qatar University, 2713 Doha, Qatar

## Abstract

Idiopathic pulmonary fibrosis (IPF) is a chronic lung disease characterized by an exacerbated fibrotic response. Although molecular and cellular determinants involved in the onset and progression of this devastating disease are largely unknown, an aberrant remodeling of the pulmonary vasculature appears to have implications in IPF pathogenesis. Here, we demonstrated for the first time that an increase of reactive oxygen species (ROS) generation induced by sera from IPF patients drives both collagen type I deposition and proliferation of primary human pulmonary artery smooth muscle cells (HPASMCs). IPF sera-induced cellular effects were significantly blunted in cells exposed to the NADPH oxidase inhibitor diphenyleneiodonium (DPI) proving the causative role of ROS and suggesting their potential cellular source. Contrary to IPF naive patients, sera from Pirfenidone-treated IPF patients failed to significantly induce both ROS generation and collagen synthesis in HPASMCs, mechanistically implicating antioxidant properties as the basis for the in vivo effect of this drug.

## 1. Introduction

Idiopathic pulmonary fibrosis (IPF) is a chronic, progressive lung disease characterized by an abnormal fibrotic response involving several areas of the lung tissue [1]. An aberrant tissue structure, encompassing exacerbated collagen secretion and deposition, progressively replaces the healthy tissue architecture, dramatically compromising the lung functions and ultimately leading to death [2]. The molecular and cellular determinants that trigger and maintain these processes are largely unknown. However, it seems that repetitive microinjuries directed towards the alveolar epithelium may play a major role [2]. Indeed, the abovementioned process leads to the release of different growth factors and fibrotic mediators such as fibroblast growth factor (FGF), platelet-derived growth factor (PDGF), and transforming growth factor-beta 1 (TGF-*β*1), which activate myofibroblast recruitment, proliferation, and accumulation of extracellular matrix in alveolar regions [2].

Although the fibroblast appears to be the most well established, other types of cells have been reported to be implicated in the IPF-associated fibrotic process [2]. In addition to a destroyed parenchyma [2], an aberrant microvascular and macrovascular remodeling of the pulmonary vasculature appears to be strongly implicated in IPF pathogenesis [3]. In this context, vascular smooth muscle cells (VSMCs) play a pivotal role in maintaining organ and tissue physiological remodeling. Indeed, under physiological conditions, the contractile phenotype of VSMCs is actively involved in the control of organ microcirculation, architecture, and function [4]. However, when a vascular injury occurs in response to proinflammatory factors, VSMCs undergo a “phenotypic switch” that confers in them the ability to proliferate, migrate, and synthesize extracellular matrix, ultimately leading to a dramatic pathological restructuring of the involved tissue [4]. Despite the massive vascular remodeling associated to IPF [3], and the potential implication of VSMCs in this process [5, 6], their role in the onset and progression of IPF-associated fibrotic phenomena remains to be elucidated. For example, whether VSMCs are involved in IPF-associated vascular remodeling in terms of increased proliferation and collagen deposition has never been investigated.

After decades of having no effective medical treatment for IPF, two recent antifibrotic agents have been introduced for the management of this pathology: Nintedanib, a potent kinase inhibitor blocking the effects of growth factors implicated in the pathogenesis of IPF (platelet-derived growth factor, vascular endothelial growth factor, and fibroblast growth factor) [7], and Pirfenidone, whose mechanisms of action are still unclear [8]. However, with Pirfenidone, some papers suggest that this molecule possesses antioxidant properties, which might account for its reported antifibrotic effect as evidenced in experimental models of lung fibrosis [9, 10]. Oxidative stress has been previously linked to IPF at both the systemic and tissue levels [11–14]. In particular, NOX-4, a ROS-generating enzyme member of the NADPH family has been reportedly implicated in IPF-associated vascular remodeling [15].

We hypothesized that prooxidant circulating factors may trigger VSMCs' phenotypic switching and induce cell proliferation and collagen I synthesis and that the antioxidant properties of Pirfenidone can counteract this phenotypic change and thus ameliorate IPF patients' conditions. To verify our two research questions, we investigated reactive oxygen species (ROS) production, cell proliferation, and collagen synthesis in primary human vascular smooth muscle cells exposed to serum obtained from naive IPF patients (IPF) or IPF patients treated with Pirfenidone (IPF + D) and healthy donors (HD).

## 2. Materials and Methods

### 2.1. Patients

IPF was diagnosticated in accordance with evidence-based guidelines for the diagnosis and management of IPF [16]. High-resolution computed tomography (HRCT) images and lung-biopsy specimens of the enrolled patients were reviewed in our hospital by two experienced radiologists and two experienced pathologists, respectively, to verify eligibility. The diagnosis for each enrolled case was approved based on a multidisciplinary discussion of experienced interstitial lung disease experts in the respiratory, pathology, and radiology departments of the University of Sassari. The eligible patients were 11.72% of the sample and had a diagnosis of consistent IPF based on the radiological pattern of usual interstitial pneumonia (UIP) on computed tomography (CT) scan, while 38% of the sample had a possible UIP pattern on CT scan; therefore, the latter was submitted to an awake surgical biopsy that showed a histological UIP pattern. In addition to the diagnosis of IPF, all enrolled cases met the following lung function criteria: percentage of predicted forced vital capacity (FVC) of at least 50% and percentage of predicted carbon monoxide diffusing capacity (DLCO) of at least 35%. All patients were treated with Pirfenidone 2403 mg/day according to the ATS ERS IPF treatment guideline [17]. Completion of a 24-week treatment period was followed by a follow-up visit 2 weeks later. Spirometry testing was performed in accordance with the criteria published by the American Thoracic Society and the European Respiratory Society [18] at baseline and after 24 weeks of treatment Table 1.

Patients with the following conditions were excluded from the study: currently in a period of acute exacerbation of IPF; comorbid conditions including malignancy, bleeding tendency, and severe hepatic dysfunction (alanine transaminase or aspartate transaminase level two-fold above the upper limit of normal, or renal serum creatinine level above the upper limit of normal); use of immunosuppressants, antifibrotic drugs including interferon, D-penicillamine, and colchicine, or oral corticosteroids at a dose ≥ 15 mg/day or the equivalent during the preceding 3 months; and current pregnancy or breastfeeding. All enrolled patients provided written informed consent, and the protocol for our study was approved by the ethics committee of the University Hospital of the University of Sassari (2262/CE-17/11/2015). Age- and sex-matched healthy donors were recruited through posted flyers and enrolled after passing a screening questionnaire aimed at excluding the presence of any underlying vascular or autoimmune disease. Blood samples were collected at the time of the IPF diagnosis and after 24 weeks of Pirfenidone treatments, and then sera were prepared according to our previously published procedures [19].

### 2.2. Cell Culture

In this study, pulmonary artery smooth muscle cells (HPASMCs) isolated from human pulmonary arteries of healthy donors were used (Innoprot, Spain). Cells were cultured in HPASMC basal medium supplemented with endothelial cell growth supplement. When confluent, HPASMCs were subcultured at a split ratio of 1 : 2 and used within three passages. Unless not specified in the text, cells were plated in 96-well black plates (BD Falcon) and processed for experiments in basal medium containing 10% (*v*/*v*) of the subjects' sera. Sera among the different subjects were normalized based on protein content. To investigate the involvement of NADPH oxidase in the IPF sera-induced cellular effects, in selected experiments, cells were pretreated for 1 hr with 10 *μ*M of the flavin-oxidase inhibitor diphenyleneiodonium (DPI) [20, 21].

### 2.3. Determination of Intracellular ROS Levels

Intracellular ROS levels were assessed by using the ROS molecular probe 2′,7′-dichlorodihydrofluorescein diacetate (H_2_DCF-DA) (Molecular Probes) as previously described [22, 23]. Within the cell, esterases cleave the acetate groups on H_2_DCF-DA, thus trapping the reduced form of the probe (H_2_DCF). Intracellular ROS oxidize H_2_DCF, yielding the fluorescent product, DCF. For ROS measurements, cultured cells were preincubated for 30 min with PBS plus containing 1 *μ*M H_2_DCFDA, then washed with PBS and treated as described. Fluorescence was measured by using a Tecan GENios Plus microplate reader (Tecan, Switzerland) in a light-protected condition. Excitation and emission wavelengths used for fluorescence quantification were 485 nm and 535 nm, respectively. Treatment-induced variation of fluorescence was kinetically measured over a time-course of 5 hours. All fluorescence measurements were corrected for background fluorescence and protein concentration. Using untreated cells as a reference, the anti- and prooxidant outcomes were evaluated by the comparison of five measurements and expressed as a means ± SD of the relative fluorescence unit (RFU) values.

### 2.4. Determination of Collagen Type I Synthesis

As previously described, collagen type-I (COLS) synthesis was investigated employing COL1A1-LV-tGFP, a GFP-based lentiviral vector (LV) driven by the human COL1A1 gene promoter [22, 24]. A red fluorescence protein-based LV (EF1*α*-LV-FP602) was used to normalize the cell transduction efficiency [22]. This method allows us to perform the real-time assessment of multiple samples at the same time in a 96-well plate using a small amount of subject sera [22]. HPMECs were transduced with lentiviral particles obtained from the pCOL1A1-LV-tGFP and EF*α*-LV-FP602 lentivectors. Transduction efficiency was checked and confirmed under a fluorescence microscope. Treatment-induced variation of fluorescence was kinetically measured over a time-course of 12 hours using a Tecan GENios Plus microplate reader (Tecan, Switzerland). Excitation wavelengths used for fluorescence quantification were 485 nm and 535 nm, while emission wavelengths were 535 nm and 590 nm for pCOL1A1-LV-tGFP and EF*α*-LV-FP602, respectively. Data were normalized for transduction efficiency by reporting the ratio of pCOL1A1-LV-tGFP to EF*α*-LV-FP602 and expressed as a means ± SD of the relative fluorescence unit (RFU) values.

### 2.5. Determination of Collagen Type I Protein Levels

Variation of collagen synthesis in response to sera treatment was quantitatively confirmed by assessing the collagen protein levels (COLP) with an enzyme immunoassay kit (Takara Bio Inc., cat. number MK101) following the protocol provided by the manufacturer. Briefly, after 48 h sera treatment cells were washed and recovered in the protein extraction buffer. 100 *μ*l of peroxidase-labeled anticollagen monoclonal antibody solution was transferred into each well for an anticollagen monoclonal antibody coating. Subsequently, 20 *μ*l of cell extract (samples were normalized for protein content) or standard was added. Then, the solution was incubated for 3 hours at 37°C. Then, 100 *μ*l of substrate solution was added into each well. The reaction between a peroxidase-labeled anticollagen monoclonal antibody and substrate results in color development with intensities proportional to the amount of collagen present in samples and standards. Finally, after 15 minutes of incubation at room temperature 100 *μ*l of Stop Solution was added into each well. The amount of collagen was quantitated by measuring the absorbance (450 nm) using a Tecan GENios Plus microplate reader (Tecan, Switzerland). Accurate concentrations of collagen in the samples were determined by comparing their specific absorbances with those obtained for the standards plotted on a standard curve. Results were expressed as a means ± SD of ng/ml collagen.

### 2.6. Determination of Cell Proliferation

Cell proliferation was assessed by using a chemiluminescent immunoassay method, which is based on the measurement of BrdU incorporation during DNA synthesis (Roche, CH). When cells are pulsed with BrdU, it is incorporated into newly synthesized DNA strands of actively proliferating cells. The incorporation of BrdU into cellular DNA can be detected using anti-BrdU antibodies, allowing assessment of the population of cells synthesizing DNA. Subconfluent cells were treated for 48 with 10% (*v*/*v*) of serum from different subjects, and BrdU was added 12 hrs before the end of the experiments. After that, the culture supernatant was removed, and the cells were fixed with a Fix-Denat solution for 30 min. The Fix-Denat was discarded and cells were incubated with an anti-BrdU antibody conjugated to horseradish peroxidase (anti-BrdU-POD) for 90 min. After rinsing three times with washing buffer, the substrate solution was added and allowed to react for 3–10 min at room temperature. Within this time window, the horseradish peroxidase catalyzes the oxidation of diacyl hydrazide, where the reaction product decaying from its excited state yields light. Finally, light emission was read by using a GENios Plus microplate reader (Tecan). Results were expressed as a means ± SD of the relative light units/sec (RLU/s) values [25, 26].

### 2.7. Statistical Analysis

Data were checked for normal distribution and processed by one-way analysis of variance (ANOVA) followed by post hoc Tukey's multiple comparison tests to determine the differences between mean values among data groups, with significance defined as *P* < 0.05. All the analyses were performed using the GraphPad Prism 6 software (GraphPad Software Inc., San Diego, CA, USA).

## 3. Results and Discussion

IPF patients enrolled in the study were predominantly males (72.72%) and had an average age of 71.27 ± 5.5 years (Table 1). At baseline (T0), patients had the following spirometry values: FVC was 81.04 ± 26.95% of the predicted value and DLCO was 54.17 ± 18.11% of the predicted value. After completion of the 24-week treatment period (T1), the mean FVC was 77.90 ± 24.49% of the predicted value and DLCO was 56.1 ± 22.38% (Table 1). Interestingly, contrary to data in the literature were IPF-untreated patients were reported to have shown a functional decline of about 120 ml at 24 weeks [23], our Pirfenidone-treated patients did not show any significant functional decline in terms of both FVC (*P* = 0.1813) and DLCO (*P* = 0.5770).

**Figure 1:**
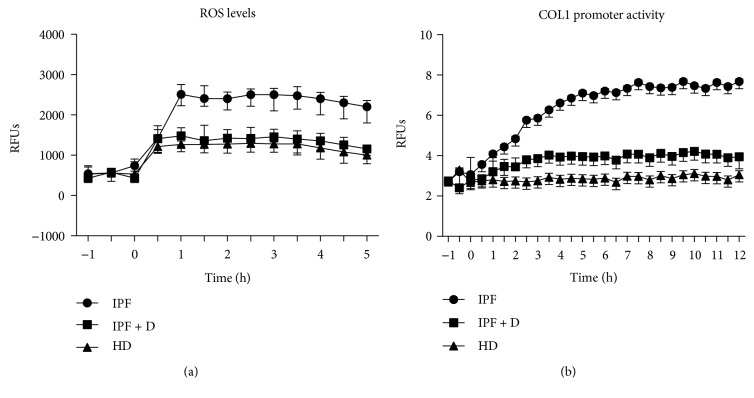
(a-b) Real-time assessment of intracellular ROS production and collagen I synthesis in HPASMCs exposed to sera of IPF patients. (a) Before stimulation, subconfluent human pulmonary artery smooth muscle cells (HPASMCs) were loaded with 10 *μ*M of H_2_-DCFDA and then cultured in basal medium containing 10% (*v*/*v*) of sera from idiopathic pulmonary fibrosis (IPF), sera from idiopathic pulmonary fibrosis patients treated for 24 weeks with Pirfenidone (IPF + D), and healthy donors (HD). Variations in intracellular ROS levels were kinetically determined in a 5-hour time-course (a randomly selected representative experiment is reported) and values at 2 hours (steady state) were used in the future comparisons. Fluorescence data were normalized for protein content and expressed as relative fluorescence units (RFUs). (b) Before stimulation, subconfluent HPASMCs were transduced with lentiviral particles obtained from the COL1A1-LV-tGFP and EF1*α*-LV-FP602 lentivectors and then cultured in basal medium containing 10% (*v*/*v*) of sera from idiopathic pulmonary fibrosis (IPF), sera from idiopathic pulmonary fibrosis patients treated for 24 weeks with Pirfenidone (IPF + D), and healthy donors (HD). Variations of COL1 promoter activation were kinetically followed for 10 hours (a randomly selected representative experiment is reported) and values at 8 hours (steady state) were used in the future comparison. Data are normalized for transduction efficiency by reporting the ratio of COL1A1-LV-tGFP to EF1*α*-LV-FP602 relative fluorescence units (RFUs).

**Figure 2:**
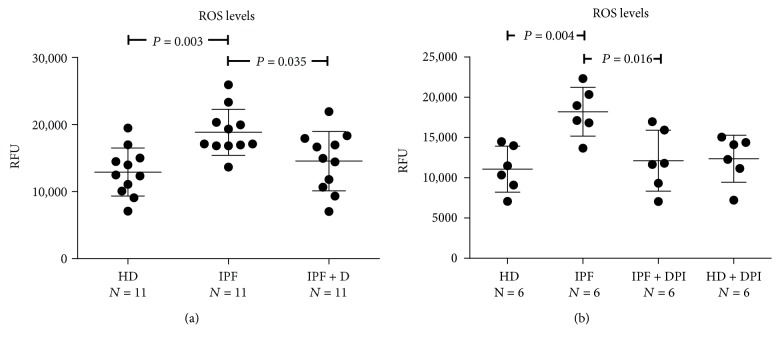
(a-b) Effects of IPF sera on HPASMC intracellular ROS levels. Before stimulation, subconfluent human pulmonary artery smooth muscle cells (HPASMCs) were loaded with 10 *μ*M of H_2_-DCFDA, then cultured in basal medium containing 10% (*v*/*v*) of sera from idiopathic pulmonary fibrosis (IPF), sera from idiopathic pulmonary fibrosis patients treated for 24 weeks with Pirfenidone (IPF + D), and healthy donors (HD). (b) In selected experiments, cells were pretreated for 60 min with the NADPH oxidase inhibitor diphenyleneiodonium (DPI) before exposure to the sera. (a-b) Data represent the variations of intracellular ROS levels after 2 hours of sera stimulation. Fluorescence data were normalized for protein content and expressed as relative fluorescence units (RFUs). *P* value indicating that the significance is reported in the figure.

Intracellular ROS levels were kinetically determined in a 5-hour time-course (Figure 1(a)) and values at 2 hours (steady state) were used for comparison (Figure 2(a)). Sera from IPF patients significantly increased intracellular ROS levels in HPASMCs compared with HD sera (Figure 2(a)). IPF-induced increase of intracellular ROS was significantly blunted by the broad NADPH oxidase inhibitor diphenyleneiodonium (DPI) [27] suggesting the involvement of the NOX family of ROS-generating enzymes in the observed surge of ROS (Figure 2(b)). As with DPI, the exposition HPASMCs to sera of Pirfenidone-treated IPF (IPF + D) patients significantly reduced the generation of intracellular of ROS elicited by IPF sera, indicating the strong antioxidant potential of this compound (Figure 2(a)).

**Figure 3:**
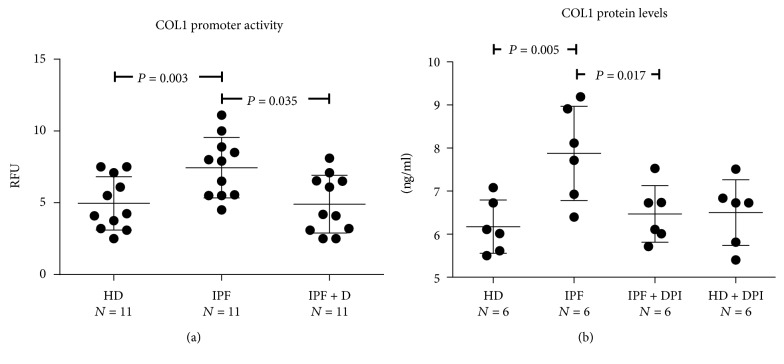
(a-b) Effects of IPF sera on HPASMC collagen I production. (a) Before stimulation, subconfluent HPASMCs were transduced with lentiviral particles obtained from the COL1A1-LV-tGFP and EF1*α*-LV-FP602 lentivectors and then cultured in basal medium containing 10% (*v*/*v*) of sera from idiopathic pulmonary fibrosis (IPF), sera from idiopathic pulmonary fibrosis patients treated for 24 weeks with Pirfenidone (IPF + D), and healthy donors (HD). Data represent the collagen I promoter activity after 8 hours of sera stimulation. Data are normalized for transduction efficiency by reporting the ratio of COL1A1-LV-tGFP to EF1*α*-LV-FP602 relative fluorescence units (RFUs). (b) Subconfluent HPASMCs were stimulated for 48 hrs with basal medium containing 10% (*v*/*v*) of sera from idiopathic pulmonary fibrosis (IPF), sera from idiopathic pulmonary fibrosis patients treated for 24 weeks with Pirfenidone (IPF + D), and healthy donors (HD) and processed for collagen I quantification as reported in the Materials and Methods. In selected experiments, cells were pretreated for 60 min with the NADPH oxidase inhibitor diphenyleneiodonium (DPI) before exposure to the sera. Data are expressed as ng/ml collagen protein. *P* value indicating that the significance is reported in the figure.

Exposure of HPASMCs to IPF sera also resulted in a progressive time-dependent increase of the collagen type 1 (COL1) promoter activity (Figure 1(b)) with values at 8 hours (steady state) significantly higher in cells exposed to IPF sera compared to HD sera (Figure 3(a)). Also in this case, the IPF-induced increase of COL1 protein expression was significantly blunted by DPI suggesting the involvement of NOX-derived ROS in the observed phenomena (Figure 3(b)). Noteworthy, similar to that observed for the ROS levels, the IPF-induced increase of COL1 synthesis was significantly attenuated when HPASMCs were exposed to sera of IPF patients treated with Pirfenidone (Figure 3(a)).

**Figure 4:**
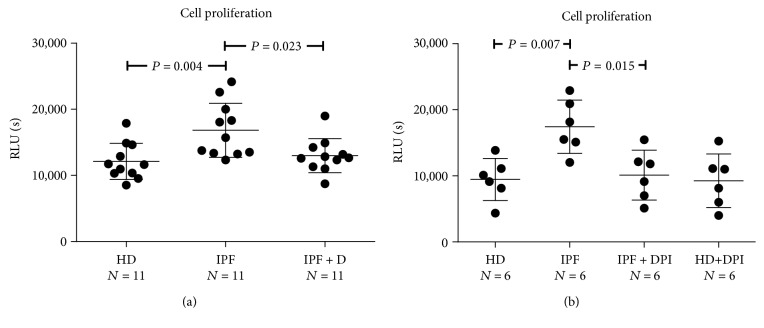
(a-b) Effects of IPF sera on HPASMC proliferation. Subconfluent HPASMCs were cultured for 48 hours in basal medium containing 10% (*v*/*v*) of sera from idiopathic pulmonary fibrosis (IPF), sera from idiopathic pulmonary fibrosis patients treated for 24 weeks with Pirfenidone (IPF + D), and healthy donors (HD). In selected experiments, cells were pretreated for 60 min with the NADPH oxidase inhibitor diphenyleneiodonium (DPI) before exposure to the sera. Data are expressed as ng/ml collagen protein. (a-b) Data are expressed as relative light units/sec (RLU/s). *P* value indicating that the significance is reported in the figure.

Data in Figure 4(a) further confirms the ability of IPF sera to elicit a HPASMC phenotypic switch in terms of increased cell proliferation, as well as the involvement of NOX-derived ROS in this phenomenon (Figure 4(b)). In addition, as previously observed for both ROS and collagen type 1, Pirfenidone significantly blunted the IPF-elicited increase of HPASMC proliferation suggesting its ability in counteracting cell function alterations elicited by prooxidant compounds present in IPF sera. Taken together, the current finding suggest that (i) phenotypic switching and collagen synthesis activation in HPASMCs may be driven by IPF sera in a ROS-dependent fashion, and that (ii) the antioxidant property of Pirfenidone may be mechanistically responsible for the observed in vivo antifibrotic effect.

Vascular remodeling in IPF fibrosis is a controversial area of research. It has now been reported that both areas with increased angiogenesis and fibrosis are present in the lungs of patients with this disease, clearly indicating aberrant microvascular tissue remodeling [28]. Indeed, many different growth factors and fibrotic mediators are released during the onset and progression of IPF, including platelet-derived growth factor (PDGF), transforming growth factor-beta 1 (TGF-*β*1), tumor necrosis factor-alpha (TNF-*α*), and vascular endothelial growth factor (VEGF) [2, 3], which play a pivotal role in the promotion of HPASMC phenotypic switching, proliferation, and eventually extracellular matrix remodeling [29–32]. TGF-*β*1 for instance, can exert its profibrotic effect via a NADPH oxidase-dependent increase of intracellular ROS levels [33]. In the lung, NADPH/NOX4-derived ROS can prompt myofibroblast activation and fibrogenic response [34]. Consistently, the expression of NADPH/NOX4 has been reportedly increased in thickened pulmonary arteries of IPF patients [6] and in pulmonary fibroblasts from IPF patients where it mediates the TGF-*β*1-induced differentiation of fibroblasts into myofibroblasts [35]. Finally, in consonance with our findings, the inhibition of NOX4 by pharmacological means reduces lung fibrosis in a rodent disease model [36].

## 4. Conclusion

This preliminary pilot study provides new evidence supporting the possibility that pathological tissue restructuring in the lung of IPF patients may be driven and/or maintained by prooxidant circulating factors acting, at least in part, through the induction of HPASMC proliferation and the activation of collagen synthesis. While more studies are needed to precisely identify both the mediators and the molecular determinants of these effects, our data provide new clues concerning the mechanism of action of Pirfenidone and indicate a rationale for considering antioxidant therapies in the treatment or prevention of IPF.

## Figures and Tables

**Table 1:** Patient demographics and clinical characteristics.

Subjects characteristics	PT0, *n* = 11	PT1, *n* = 11	HD, *n* = 11	*P* value
Age, years, mean (SD)	71.27 (5.51)	71.27 (5.51)	67.0 (9.4)	*P* = 0.21^∗^
Male, *n* (%)	8 (72.72)	8 (72.72)	9 (81.81)	*P* = 0.62^#^
Former smokers, *n* (%)	9 (81.8)	9 (81.8)	8 (72.72)	*P* = 0.62^#^
FVC, ml, mean (SD)	2343.6 (777.88)	2385.45 (801.51)		0.6328^§^
FVC, % predicted, mean (SD)	81.04 (26.95)	77.90 (24.49)		0.1813^§^
FEV_1_/FVC ratio, %, mean (SD)	92.81 (4.43)	91.66 (6.66)		0.7161^§^
DLCO, % predicted, mean (SD)	54.17 (18.11)	56.1 (22.38)		0.5770^§^

PT0, Pirfenidone T0, which refers to untreated patients just diagnosticated with IPF; PT1, Pirfenidone T1, which refers to IPF patients treated with Pirfenidone for 24 weeks; HD, healthy donors, which refers to healthy blood donors; FVC, forced vital capacity; FEV_1_, forced expiratory volume_1_; DLCO, carbon monoxide diffusing lung capacity. ^§^*P* values were determined by paired *t*-test (PT0 versus PT1), ^∗^unpaired *t*-test (HD versus PT0/PT1), and ^#^Chi-square (HD versus PT0/PT1).

## Data Availability

The data used to support the findings of this study are included within the article.

## Abbreviations

IPF: Idiopathic pulmonary fibrosis
VSMCs: Vascular smooth muscle cells
ROS: Reactive oxygen species
HPASMCs: Human pulmonary artery smooth muscle cells
HD: Healthy donors
H_2_DCF-DA: Dichlorodihydrofluorescein diacetate
COL1: Collagen type-I
LV: Lentiviral vector
tGFP: Turbo green fluorescence protein
BrdU: Bromodeoxyuridine
ECM: Extracellular matrix
FVC: Forced vital capacity
DLCO: Carbon monoxide diffusing capacity
FEV_1_: Forced expiratory volume_1_
DLCO: Carbon monoxide diffusing lung capacity.
